# Right Bundle Branch Block: A Predictor of Mortality in Early Systemic Sclerosis

**DOI:** 10.1371/journal.pone.0078808

**Published:** 2013-10-31

**Authors:** Hilda T. Draeger, Shervin Assassi, Roozbeh Sharif, Emilio B. Gonzalez, Brock E. Harper, Frank C. Arnett, Ameena Manzoor, Richard A. Lange, Maureen D. Mayes

**Affiliations:** 1 Division of Rheumatology, University of Texas Health Science Center at San Antonio, San Antonio, Texas, United States of America; 2 Division of Rheumatology and Clinical Immunogenetics, University of Texas Health Science Center at Houston, Houston, Texas, United States of America; 3 University of Texas Medical Branch at Galveston, Galveston, Texas, United States of America; 4 Division of Cardiology, University of Texas Health Science Center at San Antonio, San Antonio, Texas, United States of America; Medical University of South Carolina, United States of America

## Abstract

**Objective:**

To evaluate the prognostic significance of baseline electrocardiogram (ECG) abnormalities in a multiethnic cohort of patients with early systemic sclerosis (SSc) and to determine the serological, clinical, and echocardiogram correlates of ECG findings.

**Methods:**

SSc patients with disease duration of≤5 years were enrolled in the GENISOS (Genetics versus Environment in Scleroderma Outcome Study) cohort. At the first visit, a standard 12 lead ECG was obtained along with demographic information, clinical data, and autoantibodies. The results of echocardiograms were also recorded. All ECGs were interpreted by a cardiologist unaware of the patients' clinical data.

**Results:**

Of 265 SSc patients with average disease duration at enrollment of 2.5 years, 140 (52.8%) had abnormal ECG findings. These findings were not associated with SSc disease type or autoantibody profile but were associated with more severe heart and lung involvement. A total of 75 patients (28.3%) died over a follow up time of 9.9 years. Complete right bundle branch block (± left anterior hemiblock) on ECG, present in 7 (2.6%) patients, predicted a higher risk of mortality (HR: 5.3; 95% CI: 2.1 to 13.4; *p*<0.001). The predictive significance of right bundle branch block was independent of age at enrollment, gender, ethnicity and risk factors for coronary artery disease.

**Conclusion:**

ECG abnormalities are common in patients with early SSc and are associated with the severity of lung and heart involvement. Right bundle branch block is an independent predictor of mortality, and should be considered a marker of disease severity in SSc.

## Introduction

Cardiac involvement can occur in the limited or in the diffuse forms of systemic sclerosis (SSc) and is associated with increased mortality. [1], [2] It is often unrecognized because symptoms are nonspecific and noninvasive diagnostic techniques for early detection of cardiac involvement are not well established. [3], [4] Studies of patients with advanced SSc demonstrated electrocardiogram (ECG) abnormalities in approximately 50%. [5], [6] These abnormalities included conduction disturbances (i.e., bundle branch blocks and left anterior hemiblock), prior myocardial infarction, nonspecific ST-T wave changes, and right or left ventricular hypertrophy. [5], [6]

We have previously shown that the presence of clinically significant cardiac arrhythmias was an independent predictor of mortality in the GENISOS (Genetics versus Environment in Scleroderma Outcome Study) cohort. [7] Furthermore, ECG abnormalities are an important part of the cardiac component of Medsger Severity Index. [8] However, there are no reports on the prognostic significance of specific ECG abnormalities in early SSc.

We examined baseline ECGs in a large, multiethnic cohort of patients with early SSc to assess (a) the prevalence of ECG abnormalities; (b) their serological, clinical, and echocardiogram correlates; and (c) predictive significance of these ECG abnormalities for mortality.

## Methods

### Patient Selection

Patient data were obtained from the GENISOS cohort, a longitudinal study of patients with early SSc who were recruited within 5 years of disease onset as determined by the first non-Raynaud's phenomenon symptom. This study is a collaborative effort between the University of Texas Health Science Center at San Antonio, the University of Texas Health Science Center at Houston, and the University of Texas Medical Branch at Galveston. Recruitment into the GENISOS cohort was initiated in 1998 and continues with the enrollment of new-onset SSc cases and the follow-up of previous subjects. Patients included in the present study were recruited between 1998 and 2010. However, the vital status of enrolled patients was determined based on a death search query conducted in 2011.

Patients are eligible for enrollment if they (a) fulfill the American College of Rheumatology classification criteria for SSc or (b) had at least 3 CREST (calcinosis, Raynaud's phenomenon, esophageal dysmotility, sclerodactyly, and telangiectasias) features. [9] GENISOS is a multiethnic cohort including a sizeable number of African American and Hispanic patients.[10] The details of the GENISOS cohort are described in previous publications and are provided in Table 1. [7], [10], [11]

**Table 1:** Characteristics of patients with and without ECG abnormalities in the GENISOS Study Population.

Variable	Patients with ECG abnormality(n = 140)	Patients without ECG abnormality(n = 125)	Total(n = 265)
Age at enrollment (years), mean (±SD)	52.1±12.7	45±12.7	48.7±13.2**
Gender, female, n (%)	116 (82.9)	107 (85.6)	223(84)
Ethnicity, n (%)			
White	62 (44.3)	63 (50.4)	125 (47.2)
Hispanic	44 (31.4)	33 (26.4)	77 (29.1)
African American	33 (23.6)	21 (16.8)	54 (20.4)
Other	1 (0.7)	8 (6.4)	9 (3.4)*
History of essential hypertension, n (%)	37 (26.4)	30 (24)	67 (25.3)
History of coronary artery disease, n (%)	7 (5)	2 (1.6)	9 (3.4)
History of diabetes mellitus, n (%)	11 (7.9)	6 (4.8)	17 (6.4)
Current smoker, n (%)	22 (15.7)	23 (18.4)	45 (17.7)
Disease type, diffuse skin involvement, n (%)	76 (54.2)	73 (58.4)	149 (56.6)
Disease duration (years), mean (±SD)	2.6±1.6	2.4 ±1.6	2.5±1.6
Deceased patients, n (%)	46 (32.9)	29 (23.2)	75 (28.3)
Autoantibody profile,%			
Anti-nuclear antibody	134 (95.7)	118 (94.4)	252 (95.1)
Anti RNA Polymerase-III antibody	24 (17.1)	28 (22.4)	52 (19.6)
Anti-topoisomerase antibody	26 (18.6)	20 (16)	46 (17.4)
Anti-centromere antibody	23 (16.4)	12 (9.6)	35 (13.2)
Anti-fibrillarin Antibody	15 (10.7)	13 (10.4)	28 (10.6)
Anti U1-RNP antibody	13 (9.3)	15(12)	28 (10.6)
Anti Ro	5 (3.6)	4 (3.2)	9 (3.4)
Anti La	3 (2.1)	2 (1.6)	5 (1.9)
Myositis, n (%)	13 (9.3)	12 (9.6)	25 (9.4)
Medsger Severity Index, skin, mean (±SD)	1.6±0.8	1.5±0.8	1.6±0.8
Medsger Severity Index, lung, mean (±SD)	1.6±1.1	1.1±1	1.4±1.1**
Medsger Severity Index, heart, mean (±SD)	0.5±0.9	0.1±0.5	0.3±0.7 **
Medsger Severity Index Kidney, mean (±SD)	0.1±0.6	0.1±0.3	0.1±0.1

* p-value<0.05; ** p-value<0.001.

### Ethics Statement

The study was approved by the Institutional Review Board at University of Texas Health Science Center at San Antonio, University of Texas Health Science Center at Houston, and University of Texas Medical Branch at Galveston. All participating sites obtained written informed consents from all subjects according to the declaration of Helsinki.

### Clinical Information, Serologic Testing, and Vital Status

Age, gender, ethnicity, history of smoking, hypertension, coronary artery disease (CAD), previous stroke, SSc disease type (based on the extent of skin involvement), modified Rodnan Skin Score and disease duration were recorded for all patients. [12], [13] Disease duration was calculated from the time of onset of the first non-Raynaud's phenomenon symptom attributable to SSc. Standard 12-lead ECGs were obtained at the baseline visit. Echocardiograms were obtained within six months of performing the baseline ECG. Serum creatine kinase (CK) levels were recorded and myositis was diagnosed if the patient had proximal muscle weakness with at least one of the following: elevated levels of CK, features of myositis on electromyography, and/or a characteristic muscle biopsy. Disease severity was assessed utilizing Medsger Severity Index. [8]

For all patients, autoantibody profiles were determined at the laboratories of the University of Texas Health Science Center at Houston and the Mitogen Advanced Diagnostics at the University of Calgary. Anti-nuclear antibodies (ANA) and anti-centromere antibodies (ACA) were assayed using indirect immunofluorescence with HE*p-2* cells as the antigen substrate (Antibodies Inc., Davis, CA, USA). Passive immunodiffusion gels against calf thymus extract were used to examine sera for anti-topoisomerase-I (ATA; Scl-70), anti-Ro/SS-A, anti-La/SS-B, and anti-U1-RNP autoantibodies (INOVA Diagnostics, San Diego, California, USA). Anti-RNA polymerase III (RNAP III) was detected by enzyme-linked immunosorbent immunoassay (ELISA) kits (MBL Co. Ltd, Nagoya, Japan). Anti-fibrillarin antibodies (AFA) were determined by a line immunoassay at a serum dilution of 1∶1000 using purified recombinant fibrillarin protein (Euroline-WB: Euroimmun, Lubeck, Germany) in patients who had a positive ANA in anti-nucleolar pattern on the indirect immunofluorescence ANA.

The vital status was queried based on the National Death Index at the Center for Disease Control and Prevention, as well as the Social Security Death Index database. The date of enrollment was used as the starting point for the survival analysis.

### ECG Analysis

All ECGs were interpreted by an experienced cardiologist (RAL) who was unaware of the patients' medical condition, disease severity, and auto-antibody profile. ECG abnormalities were categorized into major and minor ECG abnormalities. [14] The following rate and rhythm characteristics were recorded: normal sinus rhythm; sinus bradycardia or tachycardia; ectopic atrial rhythm; first, second or third degree atrioventricular block; premature ventricular contractions; left or right axis deviation; delayed transition; left bundle branch block (LBBB) (complete or incomplete); right bundle branch block (RBBB) (complete or incomplete); complete right bundle branch block with left anterior hemiblock block; left or right atrial enlargement; left or right ventricle hypertrophy (LVH or RVH); non-specific ST-T wave changes; prominent U wave; ST elevation; prolonged QTc interval; low voltage; and signs indicating previous myocardial infarction.

The presence of LVH on ECG was determined by the following criteria: Sokolow-Lyon index, Cornell voltage criteria, and Romhilt and Estes voltage criteria. [15]–[17] RVH criteria were based on having right axis deviation >90 degrees and any one of the following: R:S wave ratio of ≥1 in lead V1 (in absence of posterior MI or RBBB); R wave in V1 lead>7 mm; rSR’ with R’≥10 mm; with a QRS duration of<0.12 seconds; S wave>7 mm in leads V5 or V6 (in the absence of left axis deviation>−30); RBBB with right axis deviation (axis derived from the 60 msec of QRS); RBBB with R:S waves ratio in lead I <0.5; qR wave in lead V1; S wave in leads V5 or V6>7 mm; and R:S waves ratio <1 in leads V5 or V6. If patients had RVH, right axis deviation, or RBBB, they were categorized as having ECG signs indicative of pulmonary hypertension.

### Statistical Analysis

Descriptive statistics were used to summarize the study population characteristics and the frequency of ECG findings at enrollment in SSc patients. Subsequently, the association of autoantibody profile, disease type and echocardiogram results with abnormal ECG findings were examined. For categorical variable, we used chi square (χ^2^) test or Fisher's exact test if the sample size was≤5 in one of the comparison groups. For continuous variables, t-test was used.

The impact on survival of various ECG findings at enrollment was examined by Cox regression analysis in the univariable model and after adjustment for age, gender, and ethnicity. Subsequently, we extended the multivariable Cox regression model to adjust for non-SSc related cardiac risk factors (history of hypertension, smoking, CAD, and diabetes) to examine whether the observed relationship between ECG abnormalities and survival is independent of these cardiac risk factors. In a separate multivariable model, we examined the independent predictive contribution of ECG abnormalities beyond markers of lung involvement (% predicted forced vital capacity and high right ventricular systolic pressure on echocardiogram). The statistical analyses were performed with STATA 11 (StataCorp, College Station, TX). The hypothesis testing was 2-sided with a p≤0.05 significance level.

## Results

### Population Characteristics

A total of 265 SSc patients with age 48.7±13.2 (mean±SD) years and disease duration 2.5±1.6 years at enrollment were included. Table 1 shows the details of the study population: most (84%) were female and had diffuse skin involvement (56.6%). Over the follow up period of 9.9 years, 75 patients (28.3%) died.

In the GENISOS cohort, only 125 patients (47.2%) had a normal ECG at the baseline visit. Presence of an abnormal ECG was associated with more severe lung (b = 0.48, 95% CI: 0.2–0.75, *p*<0.001) and heart (b = 0.35, 95% CI: 0.17–0.5, *p*<0.001) involvement but was not significantly related to disease duration, disease type, or serology (Table 1).

### Correlation of ECG Abnormalities with Clinical and Echocardiogram Findings

The most common ECG abnormality was nonspecific ST-T wave abnormalities (12.1%). Left or right axis deviations was detected in 18 (6.8%) and 13 (4.9%) patients, respectively. Complete RBBB and complete RBBB with left anterior hemiblock were present in 4 (1.5%) and 3 (1.1%) patients, respectively while complete LBBB was only present in 2 (0.8%) patients. Overall, 30 (11.3%) patients had ECG findings indicative of pulmonary hypertension. The frequency of ECG findings at enrollment is shown in Table 2.

**Table 2:** Frequencies of different ECG findings and their impact on mortality in the GENISOS cohort.

ECG findings	No. of patients %	Univariable Hazard Ratio (95% CI)	p value	Adjusted p value*
Normal ECG	125 (47.2)	0.59 (0.37, 0.94)	0.027	0.191
ECG findings indicative of PH	30 (11.3)	1.48 (0.76, 2.89)	0.245	0.356
**Major ECG Abnormalities**				
Previous myocardial infarction	11 (4.2)	1.54 (0.56, 4.24)	0.328	0.359
Complete left bundle branch block	2 (0.8)	N/A	1	1
Complete RBBB	4 (1.5)	5.67 (1.76, 18.26)	0.004	0.032
RBBB with left anterior hemiblock	3 (1.1)	4.38 (1.06, 18.04)	0.041	0.033
Both RBBB forms	7 (2.6)	5.3 (2.1, 13.4)	<0.001	0.003
Left ventricular hypertrophy	15 (5.7)	2.33 (1.16, 4.68)	0.017	0.239
**Minor ECG Abnormalities**				
Non-specific ST-T wave changes	32 (12.1)	1.52 (0.8, 2.89)	0.2	0.368
ST elevation	18 (6.8)	1.28 (0.56, 2.96)	0.559	0.528
Incomplete left bundle branch block	9 (3.4)	1.58 (0.5, 5.02)	0.437	0.652
Incomplete RBBB	2 (0.75)	2.07 (0.29, 14.93)	0.47	0.52
Minor QT prolongation	6 (2.3)	0.41(0.06, 2.92)	0.371	0.331
First degree atrioventricular block	14 (5.3)	1.83 (0.8, 4.23)	0.154	0.293
Left axis deviation	18 (6.8)	1.37 (0.59, 3.15)	0.462	0.977
Right axis deviation	13 (4.9)	0.77 (0.24, 2.46)	0.662	0.709
Premature atrial contractions	7 (2.6)	0.38 (0.05, 2.74)	0.25	0.222
Premature ventricular contractions	8 (3.0)	1.61 (0.39, 6.59)	0.338	0.957
Sinus tachycardia	7 (2.6)	1.7 (0.54, 5.42)	0.366	0.587
Sinus bradycardia	19 (7.2)	0.75 (0.3, 1.89)	0.794	0.551
Low voltage	20 (7.6)	1 (0.36, 2.76)	0.855	0.883
Left atrial enlargement	2 (0.8)	2.80 (0.39, 20.22)	0.307	0.376
Delayed transition	8 (3.0)	1.15 (0.28, 4.69)	0.845	0.810
Prominent U wave	4 (1.5)	1.65 (0.41, 6.75)	0.483	0.77
Right ventricular hypertrophy	3 (1.1)	1.74 (0.24, 12.53)	0.582	0.666
Right atrial enlargement	3 (1.1)	N/A	1	1
Ectopic Atrial Rhythm	3 (1.1)	2.28 (0.56, 9.31)	0.25	0.116

* p-value adjusted for age, gender, and ethnicity.

Abbreviations: PH: Pulmonary hypertension, RBBB: Right bundle branch block, N/A: Not applicable.

Table 3 summarizes the association between ECG and echocardiogram findings. At the baseline visit, 5 patients had depressed left ventricular systolic function (i.e., ejection fraction≤40%) while 20 patients had echocardiographic findings consistent with pulmonary hypertension (i.e., right ventricular peak systolic pressure >40 mm Hg). Only non-ST-T wave changes were associated with depressed left ventricular systolic function at enrollment. Non-specific ST-T wave changes and first degree atrioventricular block were significantly associated with elevated right ventricular systolic pressure on echocardiogram while RBBB and ECG findings indicative of pulmonary hypertension showed a trend for association with this echocardiographic finding.

**Table 3:** Association of ECG findings with low ejection fraction and elevated right ventricular systolic pressure on echocardiogram.

	LV-EF<40 (n = 6)	p-value	RVSP>40 mm Hg (n = 20)	p-value
**ECG findings**				
Normal ECG	0.55 (0.05, 3.96)	0.687	0.26 (0.06, 0.83)	0.018
ECG findings indicative of PH	N/A†	0.627	3.1 (0.8, 10.03)	0.052
**Major ECG Abnormalities**				
Previous myocardial infarction	N/A†	1	N/A†	0.616
Complete left bundle branch block	N/A†	1	N/A†	1
Complete RBBB	N/A†	1	6.18 (0.1, 122.16)	0.217
RBBB with left anterior hemiblock	N/A†	1	6.18 (0.1, 122.16)	0.217
Both RBBB forms	N/A†	1	6.47 (0.54, 48.1)	0.072
Left ventricular hypertrophy	N/A†	1	1.91 (0.19, 9.51)	0.329
**Minor ECG Abnormalities**				
Non-specific ST-T wave changes	8.26 (1.04, 63.83)	0.022	3.79 (1.08, 11.67)	0.019
ST elevation	3.15 (0.06, 30.7)	0.323	0.78 (0.02, 5.64)	1
Incomplete left bundle branch block	6.08 (0.11, 63.81)	0.194	3.65 (0.34, 21.02)	0.149
Incomplete RBBB	N/A†	1	N/A†	1
Minor QT prolongation	N/A†	1	N/A†	1
First degree atrioventricular block	N/A†	1	5.68 (1.16, 22.38)	0.016
Left axis deviation	2.75 (0.06, 26.57)	0.356	2.61 (0.44, 10.58)	0.154
Right axis deviation	N/A†	1	2.28 (0.23, 11.69)	0.268
Premature atrial contractions	N/A†	1	N/A†	1
Premature ventricular contractions	9.84 (0.17, 115.24)	0.133	2.44 (0.05, 23.39)	0.388
Sinus tachycardia	N/A†	1	2.03 (0.04, 18.04)	0.437
Sinus bradycardia	N/A†	1	2.81 (0.47, 11.51)	0.135
Low voltage	N/A†	1	N/A†	0.243
Left atrial enlargement	N/A†	1	N/A†	1
Delayed transition	N/A†	1	N/A†	0.659
Prominent U wave	N/A†	1	N/A†	1
Right ventricular hypertrophy	N/A†	1	6.18 (0.1, 122.16)	0.217
Right atrial enlargement	N/A†	1	N/A†	1
Ectopic Atrial Rhythm	N/A†	1	N/A†	1

** Right ventricular systolic pressure>40 mmHg; † Exact confidence levels are not possible with zero count cells Abbreviations: PH: Pulmonary hypertension, RBBB: Right bundle branch block, N/A: Not applicable.

### ECG Findings and Mortality

Table 3 shows the association of ECG findings with mortality. Patients with complete RBBB (HR: 5.67; *p* = 0.004) and RBBB with left anterior hemiblock (HR: 4.38; *p* = 0.041) had significantly higher mortality in the univariable model. As expected, a combination of these two forms of RBBB was also associated with shorter survival (HR: 5.3; *p* = 0.0008). These associations remained significant after adjustment for age, gender, and ethnicity. Presence of LVH was also associated with higher mortality in the univariable model but not in the multivariable model after adjustment for age, gender and ethnicity (*p* = 0.239).

In the extended multivariable model, the predictive significance of RBBB (combination of complete RBBB and RBBB with anterior left fascicular block) for mortality was independent of non-SSc related cardiac risk factors, namely age at enrollment, gender, ethnicity, essential hypertension, smoking, known CAD, and diabetes mellitus (HR: 3.97, 95% CI:1.48 – 10.63, *p* = 0.006). Moreover, the predictive significance of RBBB was independent of markers of pulmonary involvement. Specifically, RBBB remained a significant predictor of mortality after adjustment for age at enrollment, gender, ethnicity, % predicted FVC, presence of pulmonary hypertension on echocardiogram (HR =  3.2, 95% CI: 1.19– 8.62, *p* =  0.021)

## Discussion

The current report is the largest study to date, of the prevalence of ECG abnormalities in SSc. This study investigates for the first time the association of specific ECG abnormalities with SSc-related autoantibodies and survival.

In our multicenter study of 265 SSc patients with an average disease duration of only 2.5 years, ECG abnormalities were present in 140 (52.8%) and were equally likely to occur in those with limited or diffuse cutaneous SSc. Presence of ECG abnormalities was associated with more severe lung and heart involvement. The most common ECG abnormality in our study was nonspecific ST-T wave abnormalities. The incidence of ECG abnormalities is similar to that demonstrated in previous smaller studies of SSc patients whose disease was usually of longer duration. [5], [6] Our results suggest that cardiac involvement, as evidenced by an abnormal ECG, occurs early in SSc.

We examined the prognostic significance of ECG abnormalities in SSc patients and found that RBBB was associated with an increased risk of mortality. The predictive significance of RBBB for mortality was independent of the presence of known CAD. Presence of RBBB was not associated with higher overall or arrhythmic mortality in a large general population study [18], supporting the notion that the observed association between RBBB and mortality in the present study is secondary to SSc related lung or cardiac involvement. These findings indicate that RBBB is an early severity marker should be included in modified versions of severity indices. [8]

Previous autopsy and thallium scintigraphy data in SSc patients indicate that ventricular conduction abnormalities on ECG are a surrogate for myocardial fibrosis and are indicative of SSc cardiac involvement. [6], [19] Although RBBB in the present study showed a trend for association with presence of pulmonary hypertension on echocardiogram, the predictive significance of RBBB for mortality was independent of this echocardiographic finding and forced vital capacity. This finding raises the possibility that RBBB is either a surrogate for primary cardiac involvement (myocardial fibrosis) or undiagnosed pulmonary hypertension in SSc patients. Follow-up studies utilizing cardiac MRI in SSc patients with RBBB can differentiate between these two potential causes for this ECG abnormality.

In post-mortem studies of SSc patients, the most common histological cardiac finding was focal fibrosis randomly distributed throughout the myocardium with scattered areas of inflammation and contraction band necrosis.[20] The right and left ventricles appeared to be similarly involved, and the myocardial lesions had no relationship to coronary artery distribution (suggesting that the fibrosis was not related to a previous myocardial infarction) or to the presence of pulmonary, renal, or hypertensive disease. More than 60% of patients with severe myocardial involvement had ventricular conduction abnormalities on ECG. [20]

Although ours is the largest study examining ECG abnormalities in patients with early SSc and the first to correlate them with prognosis, it has some limitations. The study demonstrated a correlation between RBBB and increased mortality, but does not establish causation or the mechanism of death. Future longitudinal studies with noninvasive imaging (such as cardiac MRI) may further elucidate the biological basis and course of cardiac involvement in this group of SSc patients. [21] Furthermore, we could not adjust for all cardiac risk factors (specifically family history of early CAD, and dyslipidemia) because these variables were not captured in the GENISOS database. In the present study, we examined the predictors of overall mortality. In many cases, it was difficult to determine the cause of death because most patients died at outside facilities and reliable information for determining the exact cause of death was not available. Lastly, it is possible that some of the observed associations are false-positive findings; we did not correct for multiple comparisons in order to decrease the likelihood of missing clinically important associations with the investigated outcomes (β-error). However, the observed association of RBBB with mortality would also withstand Bonferroni's correction for multiple comparison (*p*_corrected_ =  0.011). Furthermore, presence RBBB and RBBB with left anterior hemiblock were both independently associated with mortality, supporting the notion that the observed association with mortality is a true biological finding rather than a random spurious result.

## Conclusion

Cardiac involvement, as evidenced by ECG abnormalities, occurs at an early stage of SSc. RBBB is an independent predictor of mortality, and should be considered a marker of disease severity in SSc. This group of patients may benefit from early cardiac and pulmonary evaluation and directed therapy.
