# Correction: Right Bundle Branch Block: A Predictor of Mortality in Early Systemic Sclerosis

**DOI:** 10.1371/annotation/f04ec0d0-8e33-487b-a619-b45ffbce6e3c

**Published:** 2014-01-06

**Authors:** Hilda T. Draeger, Shervin Assassi, Roozbeh Sharif, Emilio B. Gonzalez, Brock E. Harper, Frank C. Arnett, Ameena Manzoor, Richard A. Lange, Maureen D. Mayes

The fifth author, Brock E. Harper, was incorrectly indicated as having contributed equally to the paper with the first and second authors. 

